# IGFBP2 Modulates Trophoblast Function and Epithelial–Mesenchymal Transition in Preeclampsia via the PI3K/AKT Signaling Pathway

**DOI:** 10.3390/cimb47070478

**Published:** 2025-06-20

**Authors:** Shengping Meng, Yanping Qin, Chunyan Lyu, Sumei Wang

**Affiliations:** Department of Obstetrics, The First Affiliated Hospital of Guangxi Medical University, Nanning 530021, China; m15177321371@163.com (S.M.); qinyanping2023@163.com (Y.Q.); 13788433314@163.com (C.L.)

**Keywords:** IGFBP2, preeclampsia, invasion, EMT, PI3K/AKT, trophoblast

## Abstract

Background: Preeclampsia (PE) is a deadly obstetric complication in pregnant women leading to escalated rates of maternal and fetal mortality. Current research indicates that inadequate invasion of extravillous trophoblasts (EVTs) is a primary factor associated with the pathogenesis of PE. Insulin-like growth factor binding protein 2 (IGFBP2) plays a significant role in promoting cell migration, invasion, and angiogenesis. Researchers aim to investigate the clinical significance and elucidate the molecular mechanisms of IGFBP2 in the pathogenesis of preeclampsia. Methods: This study included 40 pregnant women categorized into 20 PE patients and 20 healthy controls. Expression levels of the mRNA were quantified using real-time quantitative polymerase chain reaction (qRT-PCR), and protein levels were assessed through Western blotting and immunofluorescence techniques. Moreover, the gain- and loss-of-function assays were conducted in human trophoblast cell line HTR-8/SVneo, and cellular models exhibiting overexpression and the knockdown of IGFBP2 were established. The proliferation, migration, and invasion of HTR-8/Svneo cells were determined using CCK8, wound-healing, and transwell assays, respectively. Results: The IGFBP2 was significantly downregulated, and the EMT was suppressed in the placental tissues of the PE patients. Functional experiments demonstrated that IGFBP2 enhanced the proliferation, invasion, and EMT of trophoblast cells activated through the PI3K/AKT signaling pathway. Conclusion: Our findings indicated that IGFBP2 enhances the proliferation, invasion, and EMT of trophoblast cells by activating the PI3K/AKT signaling pathway, serving as a potential therapeutic target in PE patients.

## 1. Introduction

Preeclampsia (PE) is a hypertensive disorder adversely affecting the health of the mothers and fetuses during pregnancy, leading to increased rates of morbidity and mortality in this group [1]. It represents approximately 8% of all gestational-related illnesses and is associated with more than 50,000 maternal deaths and 500,000 fetal fatalities worldwide. Nonetheless, the recent discovery of elevated blood pressure during pregnancy serves as the main clinical indicator of preeclampsia, characterized by systolic blood pressure (SBP) ≥ 140 mm Hg and diastolic blood pressure (DBP) ≥ 90 mm Hg. Moreover, PE can lead to various organ dysfunctions, such as impairment of the liver and kidneys, pulmonary edema, and abnormalities in the central nervous system [2]. Damage of the outer endothelial cells associated with severe preeclampsia may lead to or exacerbate cardiovascular and chronic kidney diseases. Furthermore, the progression or recurrence of preeclampsia significantly increases the risk of developing eclampsia [3]. The provision of timely prevention and therapeutic intervention can halt maternal and fetal mortality rates in preeclampsia. However, no efficient, cost-effective diagnostic strategies for this illness have been established. Previous researchers demonstrated that administering a low-dose oral aspirin in early pregnancy is the most common preventive approach for this disease [4]. In the early diagnosis of PE, elevated levels of soluble fms-like tyrosine kinase 1 (sFlt-1) in maternal serum and reduced levels of placental growth factor (PlGF) and vascular endothelial growth factor serve as essential diagnostic indicators of PE [5]. Prompt termination of the pregnancy also emerged as the most effective strategy in PE management [6]. The vast majority of studies proposed that during placental formation in PE patients, the invasiveness of extravillous trophoblast (EVT) cells is diminished, and the dysregulated uterine spiral arteries result in shallow implantation and inadequate vascular development [7]. Differentiation and invasion of EVTs, essential components in the formation of the placenta, are modulated by various factors, including cytokines, growth factors, chemokines, cell adhesion molecules, placental oxygen tension, extracellular matrix (ECM) degrading enzymes, and membrane-associated cell surface proteases [8].

The insulin-like growth factor (IGF) system consists of IGF peptides (IGF1 and IGF2), two types of IGF receptors (type I and type II), and seven families of soluble high-affinity IGF binding proteins (IGFBPs) [9]. The IGF components of the growth factor systems are essential for normal growth during embryonic and postnatal development. They play significant roles in physiological processes, such as improving the immune system functions, generating lymphocytes, and facilitating muscle differentiation and bone formation. The IGFs are essential in the cyclic development and implantation of endometrium during pregnancy [10]. This study concentrates on the insulin-like growth factor binding protein 2 (IGFBP2). It functions as a regulator of the IGF system controlling the distribution, function, and activity of IGFs in an extracellular environment [11]. Over the past decades, researchers have demonstrated that IGFBP2 expression is upregulated in solid tumors and facilitates key carcinogenic processes, including epithelial–mesenchymal transition (EMT), cell migration, invasion, angiogenesis, stem cell maintenance, transcriptional activation, and epigenetic programming through signaling pathways (independent of IGFs). Emerging research evidence indicates that abnormal expression of IGFBP2 in cancer acts is a crucial step in the carcinogenic network by integrating various cancer signaling pathways [12]. In addition to its role in cancer biology, IGFBP2 has been studied in pregnancy-associated disorders. Recent studies have demonstrated that the expression of IGFBP2 in trophoblast cells is significantly downregulated in cases of recurrent miscarriage. Furthermore, the upregulation of IGFBP2 has been shown to enhance trophoblast proliferation by activating the PI3K-AKT signaling pathway [13]. However, the expression of IGFBP2 in the serum and placental tissues of preeclamptic pregnancies remains unclear. Therefore, this study employed human tissue samples and in vitro cultured trophoblast cells to investigate the association between IGFBP2 and the pathogenesis of preeclampsia.

## 2. Materials and Methods

### 2.1. Human Tissue Collection and Ethics Statement

This study was approved by the ethics committee of the First Affiliated Hospital of Guangxi Medical University and was performed in compliance with the principles of the Declaration of Helsinki. The patients signed an informed consent before participating in this study. The clinical information, placental samples, and maternal blood samples were collected from the Department of Obstetrics at the First Affiliated Hospital of Guangxi Medical University, China. All the subjects underwent a cesarean section for pregnancy termination. The PE diagnosis was based on the criteria issued by the 2018 International Society for the Study of Hypertension in Pregnancy (ISSHP) [14]. The case group and normal pregnancy overall inclusion criteria were as follows: were aged 18–45 years; had singleton pregnancy; were within the third trimester of pregnancy; met the diagnostic criteria for preeclampsia; were free of chronic diseases (kidney disease diabetes, hypertension, or other chronic diseases), autoimmune disorders, infections, or hepatitis in preconception; and provided patient informed consent. The case group and normal pregnancy overall exclusion criteria were as follows: multiple gestations, fetal congenital malformation, fetal chromosomal disorders, history of chronic diseases, and complicated with serious internal or surgery-related disease.

Maternal venous blood samples were collected from pregnant women during their hospital stay for pregnancy termination. Maternal blood was collected into an EDTA vacuum blood collection tube and centrifuged at 3000× *g* in a 4 °C environment for 15 min. The supernatant (plasma) was extracted and stored at −80 °C for further investigations.

Within 15 min after the delivery of the placenta via cesarean section, approximately 1 cm^3^ of placental tissue was collected from a site 2 cm from the placental edge on the maternal side and as close as possible to the umbilical cord. This procedure was conducted cautiously to avoid penetrating the fetal side by avoiding large blood vessels as well as infarcted or calcified areas. The placental tissue was thoroughly washed with sterile PBS or normal saline to remove all blood and blood clots, and the samples were stored at −80 °C.

### 2.2. Cell Culture, Transduction, and PI3K/AKT Inhibitor Treatment

HTR8/SVneo cells were cultured in an RPMI-1640 medium (Gibco, Grand Island, NY, USA) and enriched with 10% fetal bovine serum (Gibco, USA) and 1% penicillin and streptomycin (Gibco, USA). The cells were incubated at 37 °C in a humidified environment with 5% carbon dioxide (CO_2_). The HTR-8/SVneo cell line was purchased from Procell (Wuhan, China).

The plasmids were extracted using a DNA Midiprep kit (Magen, Guangzhou, China). Specific plasmids were transfected using Lipofectamine 3000 (Thermo Fisher, Waltham, MA, USA). The cells were cultured in 6-well plates before transfection with the specified plasmids. The specific plasmid was transfected into the cells using an appropriate transfection reagent. Following a 24 h incubation period, the cells were harvested for subsequent experimental analysis.

A selective inhibitor of the PI3K/AKT pathway (PI3K/AKT–IN-1, MedchemExpress, Monmouth Junction, NJ, USA) was dissolved in DMSO. Following the transfection of the plasmid, the inhibitors were introduced, and the cells were maintained in culture for an additional 24 h before detecting protein pathways.

### 2.3. Plasmid Construction

The ectopic upregulation and downregulation of the IGFBP2 expression was achieved by transfecting the IGFBP2-specific plasmid (1021 bp, NM_000597, GeneChem, Shanghai, China) into the cells. The GV657 vector (GeneChem, Shanghai, China) was employed to clone the coding sequence of IGFBP2 for overexpression; the empty vector served as a negative control. In addition, the Short hairpin (sh) RNA targeting IGFBP2 (sh-IGFBP2) (GeneChem, Shanghai, China) was subsequently inserted into a plasmid vector along with the corresponding negative controls (sh-NC). The cells were collected for further experiments.

### 2.4. Quantitative Real-Time Polymerase Chain Reaction (RT-qPCR)

The RNA was extracted from the human placenta or HTR8/SVneo cells using TRIzol Reagent (Vazyme, Nanjing, China) and were reverse transcribed into cDNA with a HiScript QRT SuperMix for qPCR kit (Vazyme, Nanjing, China). The quantitative real-time PCR was performed in a 7500 Real-Time PCR System (7500, Applied Biosystems, Foster City, CA, USA) using the ChamQ SYBR qPCR Master Mix (Vazyme, Nanjing, China). The 2^−ΔΔCt^ method was employed to determine the relative mRNA levels and to normalize the relative mRNA levels to the ACTB level; the primers were used (Table 1).

### 2.5. Western Blotting

The human placental proteins were isolated using the RIPA buffer containing phosphatase and protease inhibitors (Solarbio, Beijing, China). The extraction of cellular proteins was performed using the same method described above. The proteins were separated using SDS-PAGE and were transferred to polyvinylidene fluoride membranes (Millipore, Billerica, MA, USA). These membranes were then blocked with 5% fat-free milk dissolved in TBST and incubated overnight with the corresponding primary antibodies. The samples were incubated with specific antibodies (β-actin, IGFBP2, E-cadherin, N-cadherin, Vimentin, PI3K, P-PI3K, AKT, and P-AKT) purchased from Abmart or Proteintech at a concentration of 1:1000. The Quantity One software 4.6 (Bio-Rad, Hercules, CA, USA) was employed to intensify the autoradiogram protein bands. Following this, the membranes were washed three times with the TBST buffer and incubated with a secondary antibody for 1 h (SAB, Nanjing, China). The bands were developed using the Pierce ECL Western Blotting Substrate (Tanon, Shanghai, China).

### 2.6. Immunofluorescence Assay

The HTR8/SVneo cells were fixed using 4% paraformaldehyde (Solarbio, Beijing, China) for 30 min, permeabilized with 0.5% Triton X-100 (Solarbio, Beijing, China) for 20 min and then washed in TBS several times. Following blocking with 5% bovine serum albumin (BSA) dissolved in TBS at room temperature for 30 min, the cells were incubated with primary antibodies overnight at 4 °C. On the second day, after washing the cells three times with TBST at room temperature for 1 h, they were incubated with Goat Anti-Rabbit IgG H&L (Alexa Fluor^®^ 647) secondary antibody (Abcam, Cambs, UK). The cells were incubated with DAPI staining (Solarbio, Beijing, China). The images were obtained using a fluorescence microscope (EVOS FL Aut, Waltham, MA, USA).

### 2.7. Cell Proliferation Assays

The HTR-8/SVneo transfected with mimics (3000 cells per well) were plated in 96-well plates with 5 replicates and cultured for 1 to 3 days. A total of 10 µL of CCK-8 solution (Biosharp, Beijing, China) was added to each well. The plates were then incubated at 37 °C for 2 h, and the absorbance was determined at 450 nm (A450).

### 2.8. Wound-Healing Assay

The HTR-8/SVneo transfected with the specific plasmid was re-suspended in RPMl 1640 containing 10% FBS. A 200 μL pipette tip was used to generate the wound. The cells were then cultured in a serum-free medium, and wound healing was observed at the indicated times with an inverted microscope. The photographs were captured.

### 2.9. Transwell Assays

Transwell assays were performed using Boyden chambers with 8 µm pores (Corningn, NY, USA) and Matrigel Invasion Chambers (Corningt, NY, USA). The cells were re-suspended in RPMl 1640 for serum-free and then added to the upper chamber. The lower chamber was filled with 600 μL RPMl 1640 containing 10% FBS. Following the 37 °C incubation for 48 h, the cells inside the upper chamber were extracted before the fixation. The cells on the surface of the bottom membrane were fixed with methanol and stained with 0.5% crystal violet solution (Solarbio, Beijing, China), and 3 to 5 randomly dispersed fields were counted per well.

### 2.10. Flow Cytometry Analysis of the Cell Cycle

A cell cycle staining kit (Multi Sciences, Hangzhou, China) was used to measure the cell cycle in HTR-8/SVneo. Cells were cultured in six-well plates with different treatments (specific plasmid). Phosphate-buffered saline (PBS) was used to wash the harvested cells. Then, the cells were stained with a binding buffer. The cell cycle was quantified with a FACS Vantage SE flow cytometer (BD Biosciences, San Jose, CA, USA).

### 2.11. Statistical Analysis

GraphPad Prism 9 software (Systat Software, Inc., San Diego CA, USA) was employed to analyze data obtained from three independent experiments. The results were reported in the form of mean ± SD. A one-sample *t*-test was conducted to compare the three samples to the control group. The differences among groups were determined by one-way ANOVA followed by a homogeneity of variance test. The significance level was set at *p* < 0.05.

## 3. Results

### 3.1. Clinical Characteristics

To illustrate whether the expression of IGFBP2 was changed in the PE patients, we first recruited 20 pregnant women with a normal pregnancy and 20 pregnant women with PE. No significant differences were observed in age or BMI between the two groups. However, the PE group had a significantly lower gestational age and significantly higher systolic and diastolic blood pressure as well as 24 h proteinuria compared to the normal group. Clinical data from the normal pregnant women and PE women are shown in Table 2.

### 3.2. IGFBP2 Is Down-Regulated in the Placenta of Pregnant Woman with PE, and the EMT Process Was Inhibited in the Placenta Tissues of PE

**Figure 1:**
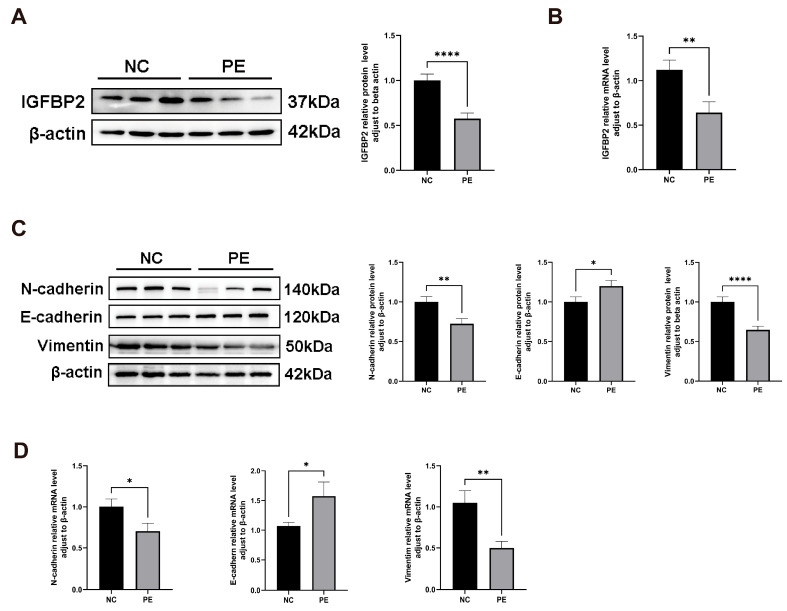
IGFBP2 is downregulated in the preeclamptic placenta, and the EMT pathway is inhibited in the preeclamptic placenta. (**A**) A Western blot analysis was used to determine the IGFBP2 protein level and EMT-related protein regarding E-cadherin, N-cadherin, and Vimentin in preeclamptic or normal pregnancy placentae (*n* = 20). (**B**) An RT-PCR analysis was used to determine IGFBP2 mRNA levels in preeclamptic or normal pregnancy placentae (*n* = 20). (**C**) A Western blot analysis was used to determine the EMT-related protein regarding E-cadherin, N-cadherin, and Vimentin in preeclamptic or normal pregnancy placentae (*n* = 20). (**D**) An RT-PCR analysis of EMT-related genes about E-cadherin, N-cadherin, and Vimentin mRNA level in preeclamptic or normal pregnancy placentae (*n* = 20). PE represents the patients with preeclampsia pregnancies. NC represents individuals with normal pregnancies. The data is expressed as mean ± SD. * indicates *p* < 0.05, ** indicates *p* < 0.01, **** indicates *p* < 0.0001.

To investigate the role of IGFBP2 in PE, we compared its expression levels in placental tissues from normal and PE pregnant women. IGFBP2 mRNA and protein levels were analyzed by RT-qPCR and a Western blot. IGFBP2 levels were significantly lower in the PE group than in the NC group (Figure 1A,B).

Similarly, EMT biomarker levels of E-cadherin, N-cadherin, and Vimentin were detected by RT-qPCR and a Western blot. E-cadherin mRNA and protein levels were significantly higher, while N-cadherin and Vimentin levels were significantly lower in preeclamptic placentas (Figure 1C,D).

### 3.3. Knockdown of IGFBP2 Inhibited Growth, Invasion, and EMT of HTR-8/SVneo Cells

**Figure 2:**
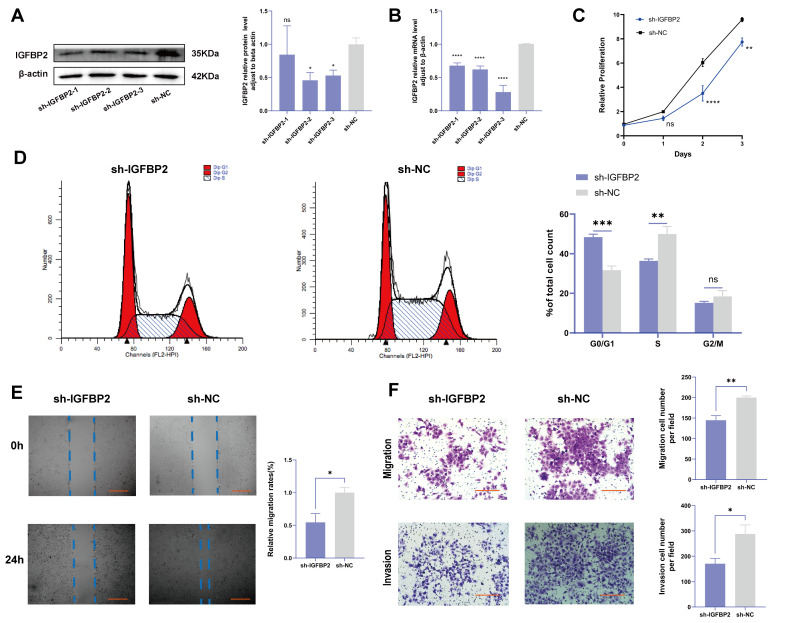
The knockdown of IGFBP2 inhibited growth and the invasion of HTR-8/SVneo cells. HTR-8/SVneo cells were transfected with shRNAs against IGFBP2. (**A**,**B**) Western blotting and RT-qPCR were used to assess the knockdown efficiency. (**C**) The CCK-8 assay was used to detect the proliferation of HTR8/SVneo cells in each group. (**D**) Flow cytometry was employed to analyze the cell cycle distribution in cells with IGFBP2 knockdown. (**E**) The wound-healing assay was used to examine the migratory capabilities of HTR8/SVneo cells in each group; scale bar = 1000 μm. (**F**) The transwell assay was used to examine the migratory and invasive capabilities of HTR8/SVneo cells in each group; scale bar = 200 μm, *n* = 3. The data is expressed as mean ± SD. An independent sample *t*-test was used to compare the two groups. The two-way ANOVA and the Bonferroni post hoc analysis were used to compare different time intervals of the data. ns indicates no significant difference. * indicates *p* < 0.05, ** indicates *p* < 0.01, *** indicates *p* < 0.001, **** indicates *p* < 0.0001.

**Figure 3:**
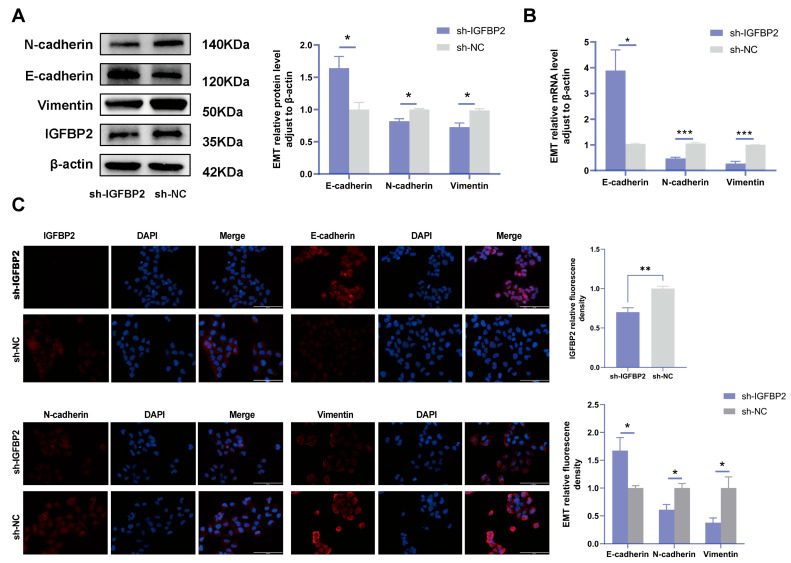
IGFBP2 knockdown inhibited the EMT of HTR-8/SVneo cells. The HTR-8/SVneo cells were transfected with shRNAs targeting IGFBP2. (**A**,**B**) Western blotting and RT-qPCR were used to assess the EMT levels of biomarkers E-cadherin, N-cadherin, and Vimentin. (**C**) Immunofluorescence was used to determine the relative expression levels of E-cadherin, N-cadherin, and Vimentin; scale bar = 100 μm, *n* = 3. The data is expressed as mean ± SD. An independent sample *t*-test was conducted to compare data across the groups. The two-way ANOVA and the Bonferroni post-hoc analysis were used to compare different time intervals across the groups. * indicates *p* < 0.05, ** indicates *p* < 0.01, *** *p* < 0.001.

To investigate the biological effects of IGFBP2 on trophoblast cells, we synthesized three specific shRNA fragments inserted into a plasmid vector to reduce the expression of exogenous IGFBP2 in HTR-8/SVneo cells. Moreover, Western blotting and qRT-PCR analyses were employed to assess the transfection efficiency for the specific shRNAs plasmid. The results indicated that three IGFBP2 decreased the protein expression of IGFBP2 compared to sh-NC, with sh-IGFBP2-3 revealing a more efficient knockdown than sh-IGFBP2-1 and sh-IGFBP2-2 (Figure 2A,B). Therefore, sh-IGFBP2-3 was used in subsequent investigations and was designated sh-IGFBP2. Subsequently, wound-healing and transwell assays were performed to assess the role of IGFBP2. Notably, the CCK8 effectively inhibited cell proliferation in cells containing sh-IGFBP2 plasmids (Figure 2C). The cell cycle was analyzed by flow cytometry, and the results indicated that the downregulation of the IGFBP2 gene significantly influenced cell cycle progression by increasing the number of cells in the G1 phase and decreasing those in the S/G2 phases (Figure 2D). The results of the wound-healing assay revealed that IGFBP2 downregulation reduced the migratory capabilities of cells (Figure 2E). Moreover, the results of the transwell assay depicted that IGFBP2 downregulation suppressed the migration and the invasion abilities of the cells (Figure 2F). Cells were evaluated to assess the expression levels of biomarkers associated with EMT. The results showed that the levels of N-cadherin and Vimentin were substantially lower than that of sh-NC. Furthermore, cells with sh-IGFBP2 exhibited relatively high levels of E-cadherin compared to cells transfected with sh-NC (Figure 3A,B). Additionally, we used immunofluorescence methods to corroborate these findings (Figure 3C).

### 3.4. Overexpression of IGFBP2 Promoted Growth, Invasion, and EMT of HTR-8/SVneo Cells

**Figure 4:**
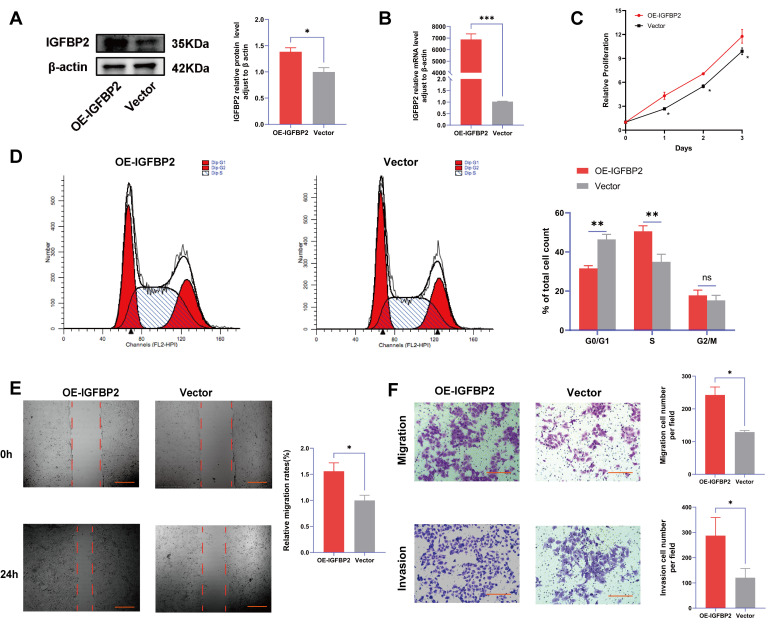
IGFBP2 overexpression promoted the growth and invasion of HTR-8/SVneo cells. The HTR-8/SVneo cells were transfected with IGFBP2 overexpression plasmids to upregulate the expression levels of IGFBP2. (**A**,**B**) Western blotting and RT-qPCR were used to assess the upregulation efficiency. (**C**) The CCK-8 assay was used to detect the proliferation of HTR8/SVneo cells in each group. (**D**) Flow cytometry was employed to analyze the cell cycle distribution in cells with IGFBP2 overexpression. (**E**) The wound-healing assay was used to examine the migratory capabilities of HTR8/SVneo cells in each group; scale bar = 1000 μm. (**F**) The transwell assay was used to examine the migratory and invasive capabilities of HTR8/SVneo cells in each group; scale bar = 200 μm. Data was expressed as mean ± SD, (*n* = 3). An independent sample *t*-test was used to compare the data between the two groups. The two-way ANOVA and Bonferroni post-hoc test were utilized to compare the data at different time intervals. ns indicates no significant difference. * indicates *p* < 0.05, ** indicates *p* < 0.01, *** indicates *p* < 0.001.

**Figure 5:**
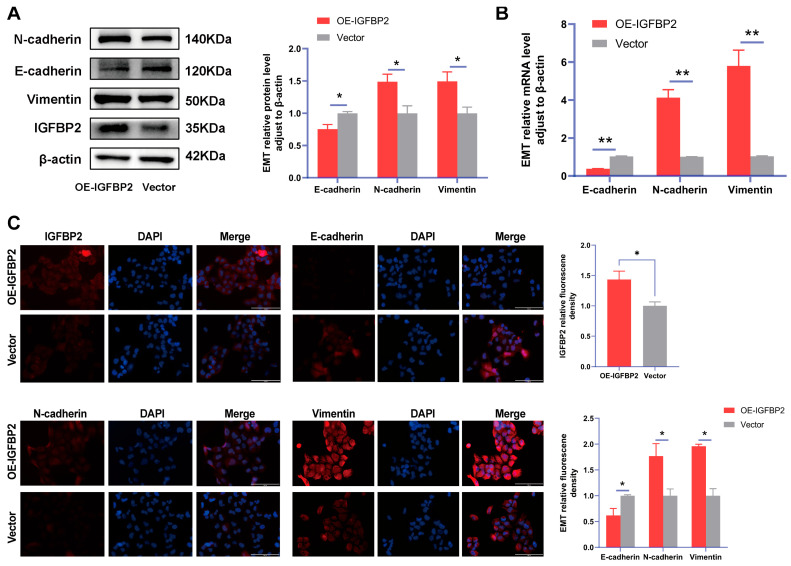
IGFBP2 overexpression of promoted EMT of HTR-8/SVneo cells. The HTR-8/SVneo cells were transfected with IGFBP2 overexpression plasmids to upregulate IGFBP2 expression levels. (**A**,**B**) Western blotting and RT-qPCR were used to assess the EMT biomarkers levels of E-cadherin, N-cadherin, and Vimentin were assessed. (**C**) Immunofluorescence was used to determine the relative expression levels of E-cadherin, N-cadherin, and Vimentin (scale bar = 100 μm) (*n* = 3). The data was expressed as the mean ± SD. An independent sample *t*-test was used to compare data across two groups. * indicates *p* < 0.05, ** indicates *p* < 0.01.

To further verify the function of IGFBP2 in the progression of PE, the gain-of-function experiments were conducted by transfecting cells with IGFBP2 overexpression plasmids. Following this transfection, the levels of IGFBP2 were elevated (Figure 4A,B). The CCK8 assay revealed that cell proliferation was promoted in cells treated with IGFBP2 overexpression plasmids (Figure 4C). Additionally, flow cytometry analyses indicated that the overexpression of IGFBP2 significantly influenced the progression of the cell cycle (Figure 4D). In the wound-healing assay, the cells that overexpressed IGFBP2 exhibited a relatively faster migration rate than the control cells (Figure 4E). In the transwell assay, the number of migrated and invaded cells was higher in the IGFBP2-overexpressing group than in the control group (Figure 4F). The experiment revealed that IGFBP2 treatment facilitated the migratory and invasive capabilities of the cells. The expression levels of EMT-related molecules in cells overexpressing IGFBP2 were assessed using RT-qPCR and Western blot analyses (Figure 5A,B). The results indicated that the upregulation of IGFBP2 led to a significant downregulated E-cadherin compared to the control group, while N-cadherin and Vimentin were upregulated. Immunofluorescence results corroborated these results. (Figure 5C).

### 3.5. IGFBP2 Promoted Growth, Invasion, and EMT of HTR-8/SVneo Cells by Activating PI3K/AKT Pathway

**Figure 6:**
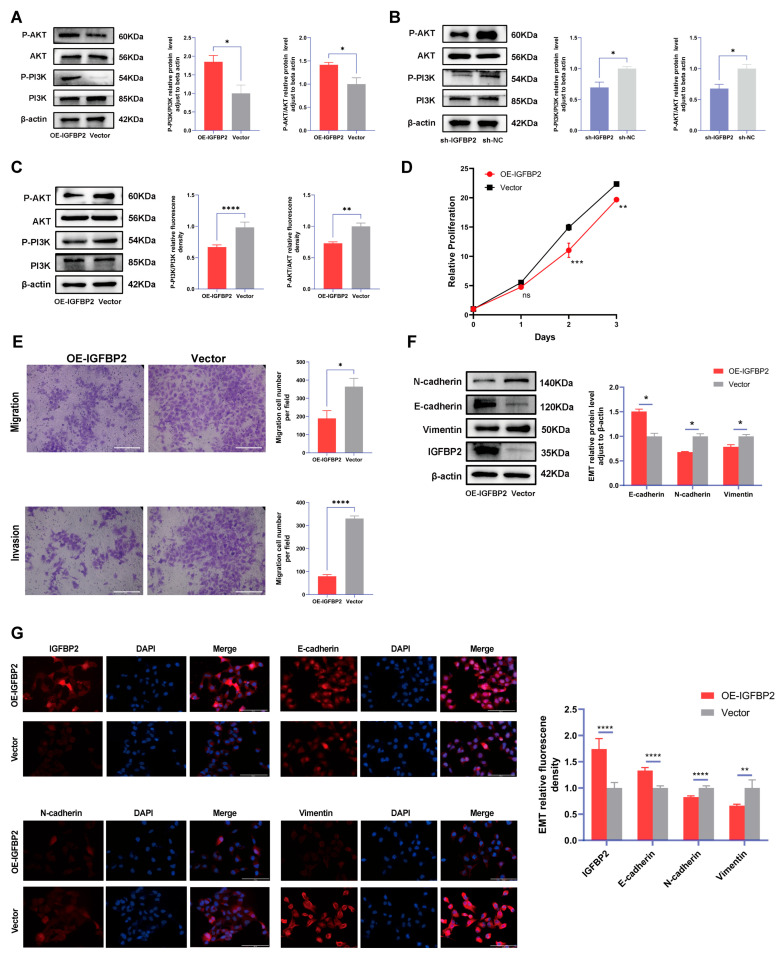
The function of IGFBP2 is mediated via the PI3K/AKT pathway. (**A**) The HTR-8/SVneo cells were transfected with IGFBP2 overexpression plasmids to upregulate IGFBP2 expression levels. The expression of the PI3K-AKT signaling pathway-related proteins P-PI3K/PI3K and P-AKT/AKT in HTR-8/SVneo cells detected by the Western blot analysis. (**B**) The expression levels of PI3K-AKT signaling pathway-related proteins, including P-PI3K/PI3K and P-AKT/AKT, were quantitatively analyzed in HTR-8/SVneo cells with IGFBP2 knockdown using the Western blot analysis. (**C**–**G**) The HTR-8/SVneo cells were transfected with IGFBP2 overexpression plasmids and incubated with 20μM PI3K/AKT–IN-1 for 24 h. (**C**) The relative protein expression of P-PI3K/PI3K and P-AKT/AKT was determined by the Western blot with the management of PI3K/AKT–IN-1 in HTR-8/SVneo cells. (**D**) The CCK-8 assay was used to detect the proliferation of HTR8/SVneo cells in each group after the management of PI3K/AKT–IN-1 in HTR-8/SVneo cells. (**E**) The transwell assay was used to examine the migratory and invasive capabilities of HTR8/SVneo cells in each group; scale bar = 200 μm. (**F**) Western blotting was used to assess the EMT biomarkers levels of E-cadherin, N-cadherin, and Vimentin with the management of PI3K/AKT–IN-1 in HTR-8/SVneo cells. (**G**) The immunofluorescence was used to determine the relative expression levels of E-cadherin, N-cadherin, and Vimentin with the management of PI3K/AKT–IN-1 in HTR-8/SVneo cells. (scale bar = 100 μm). The data was expressed as the mean ± SD. An independent sample *t*-test was used to compare data across two groups. The two-way ANOVA and Bonferroni post-hoc test were utilized to compare the data at different time intervals. ns indicates no significant difference. * indicates *p* < 0.05, ** indicates *p* < 0.01, *** indicates *p* < 0.001, **** indicates *p* < 0.0001.

To explore the role of IGFBP2 in the progression of PE, the expression of the PI3K/AKT pathway, a classical pathway integrated with the functions of IGFBP2, was examined in cells. The IGFBP2 knockdown significantly reduced protein levels of p-PI3K/PI3K and p-AKT/AKT, whereas IGFBP2 overexpression substantially enhanced expression levels of p-PI3K/PI3K and p-AKT/AKT in HTR-8/SVneo cells (Figure 6A). Subsequently, PI3K/AKT–IN-1 were administered to cells and overexpressed IGFBP2. The results indicated that the inhibitor relatively reduced the levels of p-PI3K/PI3K and p-AKT/AKT (Figure 6B). The CCK8 assay revealed that cell proliferation was inhibited in cells treated with pathway inhibitors (Figure 6C). In the transwell assay, the number of migrated and invaded cells were suppressed in the IGFBP2-overexpressing group than in the control group treated with pathway inhibitors (Figure 6D,E). Additionally, Western blotting and immunofluorescence assays were employed to demonstrate that the relative protein expression of E-cadherin was reduced and the relative protein expression of N-cadherin and Vimentin was increased (Figure 6F,G).

## 4. Discussion

PE is a condition distinctively associated with elevated blood pressure in pregnancy. Previous researchers indicated that PE primarily originates in the placenta [15]. Therefore, investigating fundamental molecular mechanisms underlying placental trophoblast dysfunction can provide vital insights for PE diagnosis and management. EMT presents a process whereby epithelial cells lose their polarity and adhesion, transforming into a mesenchymal phenotype and acquiring enhanced migratory capacity. The key molecular markers of EMT include the loss of E-cadherin and the acquisition of N-cadherin and Vimentin [16]. This study identified that the IGFBP2 expression downregulation and EMT process was inhibited in the placental tissue of PE patients, suggesting that IGFBP2 and EMT play an important role in the development of preeclampsia. Therefore, the downregulation of IGFBP2 expression in the placental tissues of patients with preeclampsia may be associated with impaired invasive capacity of trophoblast cells and may lead to abnormal placental development by inhibiting the EMT process, thereby contributing to the pathogenesis of preeclampsia.

To elucidate the impact of IGFBP2 on trophoblast invasion, HTR-8/SVneo cell lines with IGFBP2 overexpression or knockdown were established, and the role of IGFBP2 in the biological functions was examined. The findings indicated that upregulating IGFBP2 enhanced the proliferation, migration, invasion capabilities, and EMT of trophoblast cells, whereas the knockdown of this protein inhibited these processes. The IGFBP2 promoted the activation of the PI3K/AKT pathway and mediated the role of IGFBP2 in cell growth, invasion, and EMT of HTR-8/SVneo cells. To the best of our knowledge, this is the first study to determine the roles of IGFBP2 and the downstream mechanisms of IGFBP2 in PE.

Research indicates that IGFBP2 is significantly expressed in rapidly proliferating cell populations, with increased expression levels observed near cellular growth and differentiation, highlighting its essential role in the development of fetal tissues [17]. The IGFBP2 may be a potential biomarker for predicting the risk of PE development. Therefore, this study systematically clarified the specific expression patterns of IGFBP2 in placental tissue and explored its significance in the potential mechanisms and placental malformation.

Previous studies demonstrated that abnormal expression patterns of IGFBP2 are detectable in various human cancers and are significantly associated with poor prognosis. For example, research on breast cancer reported that the expression levels of IGFBP2 were substantially elevated in the T1 stage of breast cancer compared to benign lesions. This suggests that IGFBP2 can potentially facilitate metastatic development and serve as a critical biomarker for predicting lymph node metastasis in breast cancer [18]. Further studies on melanoma identified IGFBP2 as a direct downstream target of MDA-9/Syntenin, which regulates endothelial cell proliferation, migration, and invasion. This involvement facilitates tumor angiogenesis, tumor initiation, and progression [19]. Therefore, IGFBP2 can potentially modulate the invasive capabilities of diverse cell types.

The onset of PE is significantly associated with a reduction in the invasive capacity of trophoblast cells. Therefore, preserving the invasive potential of these cells can effectively alleviate this disease. In this study, the loss- and gain-of-function results demonstrated that IGFBP2 increased cell viability and invaded cell numbers. The IGFBP2 overexpression facilitated the EMT process. Conversely, the IGFBP2 downregulation generated the opposite effect. Similarly, the findings indicated that IGFBP2 is reduced in the placental tissues derived from preeclampsia placental tissue related to EMT inhibition. The phenotypic transitions between epithelial and mesenchymal states, particularly EMT and mesenchymal–epithelial transition (MET), are essential for the intricate remodeling of embryonic and organ structures during gastrulation, organogenesis, and the metastatic progression of various cancers [20]. The motility and invasive phenotype of EVTs during placental development are associated with the EMT process [21]. Therefore, our results further indicate that impaired invasive capacity of trophoblast cells attributed to IGFBP2 downregulation could be linked to suppressed EMT.

Additionally, we identified that the overexpression of IGFBP2 enhanced the PI3K/AKT pathway levels in HTR-8/SVneo cells. The PI3K/AKT inhibitors effectively induced proliferation and decreased the migration and invasion of HTR-8/SVneo cells. This denotes that the PI3K/AKT pathway may have therapeutic potential in treating HTR-8/SVneo cells. In a previous study on the investigation of the occurrence and progression of recurrent spontaneous abortion transcriptome sequencing, the results indicated that IGFBP2 could modulate the biological behavior of trophoblast cells through the PI3K/AKT signaling pathway and positively impact pregnancy outcomes [13]. Additionally, previous studies strongly associated IGFBP2 and the PI3K/AKT pathway [22,23]. Previous research demonstrated that the PI3K/AKT signaling pathways are essential for trophoblast function in various molecular cascades associated with PE. The downstream targets of these pathways encompass cellular processes, such as migration and invasion [24]. It is currently unclear whether downstream targets in this pathway are linked to IGFBP2 and whether IGFBP2 facilitates EMT through alternative mechanisms. For example, classical TGF-β signaling, ERK, MAPK, and PI3K-AKT collaboratively play a crucial role in inducing EMT across different tissue types [25]. These signaling pathways distinctively facilitate the expression of EMT processes in distinct manners [16]. Furthermore, IGFBP2 is capable of inducing excessive activation of its biological functions in cancer via multiple signaling pathways. For example, in hepatocellular carcinoma, IGFBP2 facilitates the EMT process by activating the Wnt/β-catenin signaling pathway [26]. Our findings indicate that IGFBP2 enhances the proliferation, invasion, and EMT of trophoblast cells via activating the PI3K/AKT signaling pathway, potentially offering a novel therapeutic target for PE. Understanding the mechanisms of abnormal placentation could advance our current knowledge of the pathogenesis of pre-eclampsia and other disorders. The limitations of our study are mainly related to a relatively small sample size of participants having PE. The main findings of this study indicate that the expression of IGFBP2 in the placenta was obtained from small sample sizes, necessitating cautious interpretations of the differences in IGFBP2 expression in the population. This study specifically examined expression levels of IGFBP2 in placenta during the third trimester of pregnancy. However, the underlying characteristics of its variation during the first and second trimesters remain to be elucidated. Despite considerable limitations in the current findings, the results of this study are relevant and add to the evidence on the subject matter. Future studies could further investigate expression profiles of IGFBP2 during early pregnancy and evaluate the potential utility of measuring IGFBP2 levels in early gestation for predicting preeclampsia.

## 5. Conclusions

In summary, the activation of the IGFBP2-mediated PI3K/AKT signaling pathway enhances the proliferation, migration, invasion, and EMT of trophoblast cells in preeclampsia. The findings of this study indicate that IGFBP2 may play a critical role in the pathogenesis of preeclampsia, thereby offering a novel avenue for further investigation into the underlying mechanisms of this condition.

## Figures and Tables

**Table 1:** The sequences of the primers.

Name	Forward (5′-3′)	Reverse (5′-3′)
IGFBP2	AACAGTGCAAGATGTCTCTGAACGG	GCCTCCTGCTGCTCATTGTAGAAG
N-cadherin	ATCAAGCCTGTGGGAATCCG	CTCTATGGGCCAGGTTTTCTCA
E-cadherin	GCTGGACCGAGAGAGTTTCC	CAAAATCCAAGCCCGTGGTG
Vimentin	CCTCCGGGAGAAATTGCAGG	GCGTTCAAGGTCAAGACGTG
β-actin	CCTTCCTGGGCATGGAGTC	TGATCTTCATTGTGCTGGGTG

**Table 2:** Clinical characteristics of normal patient group and preeclampsia patient group.

Variable	Normal Pregnancy	Preeclampsia	*p*-Valve
Number	20	20	
Maternal age (years)	34.55 ± 4.395	32.85 ± 6.409	>0.05
Gestational age (weeks)	38.73 ± 1.086	35.51 ± 3.023	<0.05
BMI (kg/m^2^)	27.72 ± 3.294	29.29 ± 4.531	>0.05
Systolic BP (mmHg)	116.60 ± 10.081	156.45 ± 18.346	<0.05
Diastolic BP (mmHg)	71.60 ± 10.210	97.65 ± 16.294	<0.05
Proteinuria	−	+ to +++	<0.05
Birth weight (g)	3371.00 ± 558.135	2223.50 ± 945.310	<0.05

“−” indicates negative urine protein or not tested. “+” indicates different degrees of severity of 24-h urine protein.

## Data Availability

The data will be made available on request.
